# Radical surgery for intractable thoracic empyema complicating traumatic pneumothorax and rib fractures

**DOI:** 10.1186/s40792-023-01765-x

**Published:** 2023-10-24

**Authors:** Teppei Tokumaru, Hideaki Kurata, Jin Mitsui, Joji Tomioka

**Affiliations:** 1 Emergency and Critical Care Center, Yamaguchi Prefectural Grand Medical Center, Osaki, Hofu City, Yamaguchi 10077747-8511 Japan; 2 Department of Acute Medicine and Surgery, Yonemori Hospital, 1-7-1 Yojiro, Kagoshima City, Kagoshima 890-0062 Japan

**Keywords:** Antibiotic prophylaxis, Rib fracture, Pneumothorax, Thoracic empyema, Rib fixation, Lung decortication

## Abstract

**Background:**

Few cases of traumatic pneumothorax complicated by thoracic empyema have been reported. The indication of antibiotic prophylaxis administration for traumatic pneumothorax during tube thoracostomy remains controversial, and thoracic injury complicated by empyema can be life-threatening and intractable.

**Case presentation:**

A 42-year-old male patient was injured during a collision with a passenger car while driving a motorcycle. The patient (body mass index, 37 kg/m^2^) was diagnosed with right first-to-sixth-rib fractures without a flail segment, right clavicle fracture, and slight hemopneumothorax. Tube thoracostomy was performed for traumatic pneumothorax on day 3 without antibiotic prophylaxis. The patient demonstrated a progressive displaced rib fracture complicated by empyema on day 11. Radical surgery was performed for the empyema with rib fixation on day 15. The postoperative course was uneventful, and the patient was discharged from the hospital on day 31.

**Conclusions:**

A traumatic pneumothorax can be complicated by empyema. Thoracic injuries complicated by empyema can be life-threatening and intractable. Antibiotic prophylaxis for traumatic pneumothorax with tube thoracotomy should therefore be considered in select cases. The strategy for thoracic injury requires the assumption of an occult thoracic infection and chest wall instability.

## Background

Thoracic injuries, including pneumothorax and rib fractures, can often be successfully treated using non-operative management. During the management of such injuries, thoracic injuries complicated by respiratory failure and infection can become life-threatening and intractable [1].

Traumatic pneumothorax is a risk factor for thoracic empyema; however, few cases of traumatic pneumothorax complicated by thoracic empyema have been reported [2]. The indication for antibiotic prophylaxis for blunt traumatic pneumothorax in tube thoracostomy remains controversial [3–6]. The efficacy of early surgical fixation for rib fractures is currently being debated [7]; therefore, cases of thoracic injury with infection should also be discussed.

Herein, we report a case of traumatic pneumothorax complicated by empyema that was successfully treated with radical surgery utilizing posterolateral thoracostomy for life-threatening and intractable thoracic empyema and rib fractures. No similar cases have been reported; therefore, the strategies and tactics for such clinical cases should be discussed.

## Case presentation

**Fig. 1:**
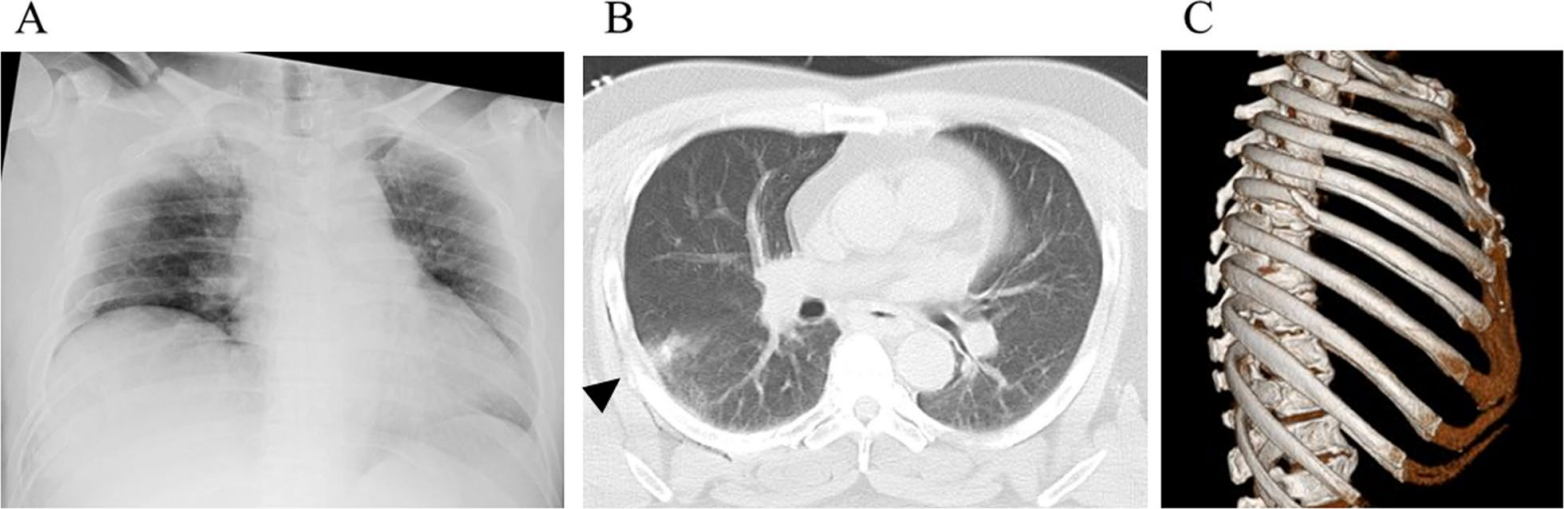
**A** Chest X-ray shows the first to sixth ribs and clavicle fractures with hemopneumothorax. **B** Chest computed tomography revealed pulmonary contusion below the right sixth rib fracture (black arrowhead). **C** The rib fractures demonstrated no flail segment

A 42-year-old male patient was injured in a collision with a passenger car while driving a 250-cc motorcycle. The diagnosis on admission was right first-to-sixth-rib fractures without a flail segment and chest wall instability, right clavicle fracture, and superficial pulmonary contusion with slight hemopneumothorax (body mass index, 37 kg/m^2^) (Fig. 1). The patient underwent non-operative management without tube thoracostomy. Pneumothorax with subcutaneous emphysema became apparent on day 3 of admission. As a result, tube thoracostomy was performed; the catheter size was 20 Fr, and the suction pressure was set at − 10 cmH_2_O. Antibiotic therapy was not administered to the patients before or after the course. Chest tube management was continued for minor air leakage from pneumothorax.

**Fig. 2:**
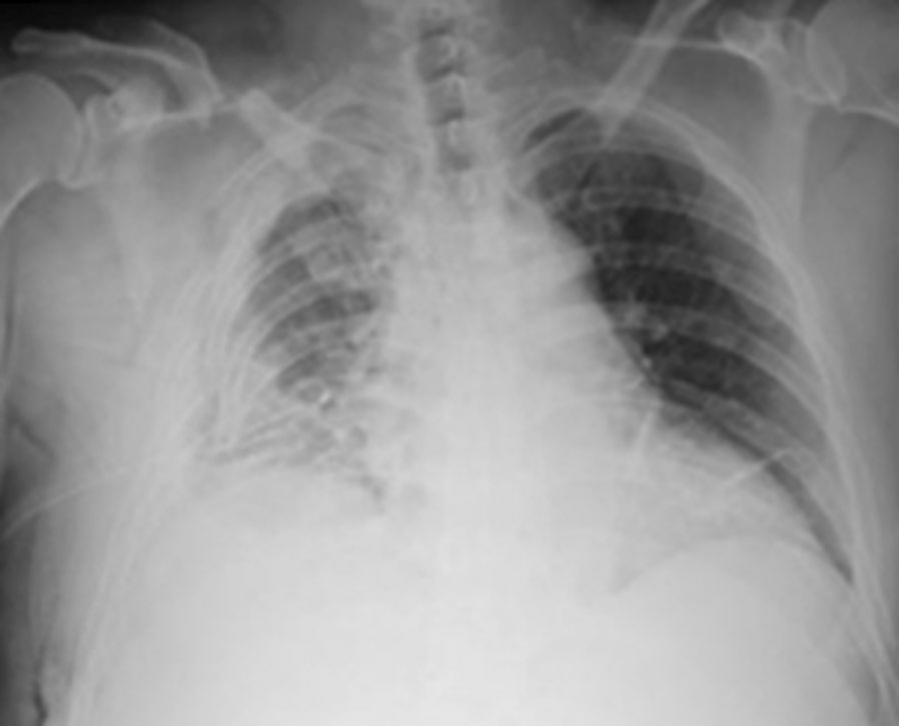
Chest X-ray showed advanced chest wall destruction with rib fractures and a collapsed lung

The sudden onset of acute respiratory failure led to the implementation of noninvasive positive-pressure ventilation management on day 11. Until then, the patient’s general condition had been satisfactory without fever, and chest tube drainage revealed clear fluid. Chest radiography revealed advanced destruction of the chest wall, rib fractures, and an unexpandable lung. The displaced rib fracture was evident and exacerbated, destroying the right chest wall (Fig. 2). Laboratory findings included a white blood cell count of 22,500/µL and a C-reactive protein level of 14.26 mg/dL; thus, thoracic empyema was suspected.

**Fig. 3:**
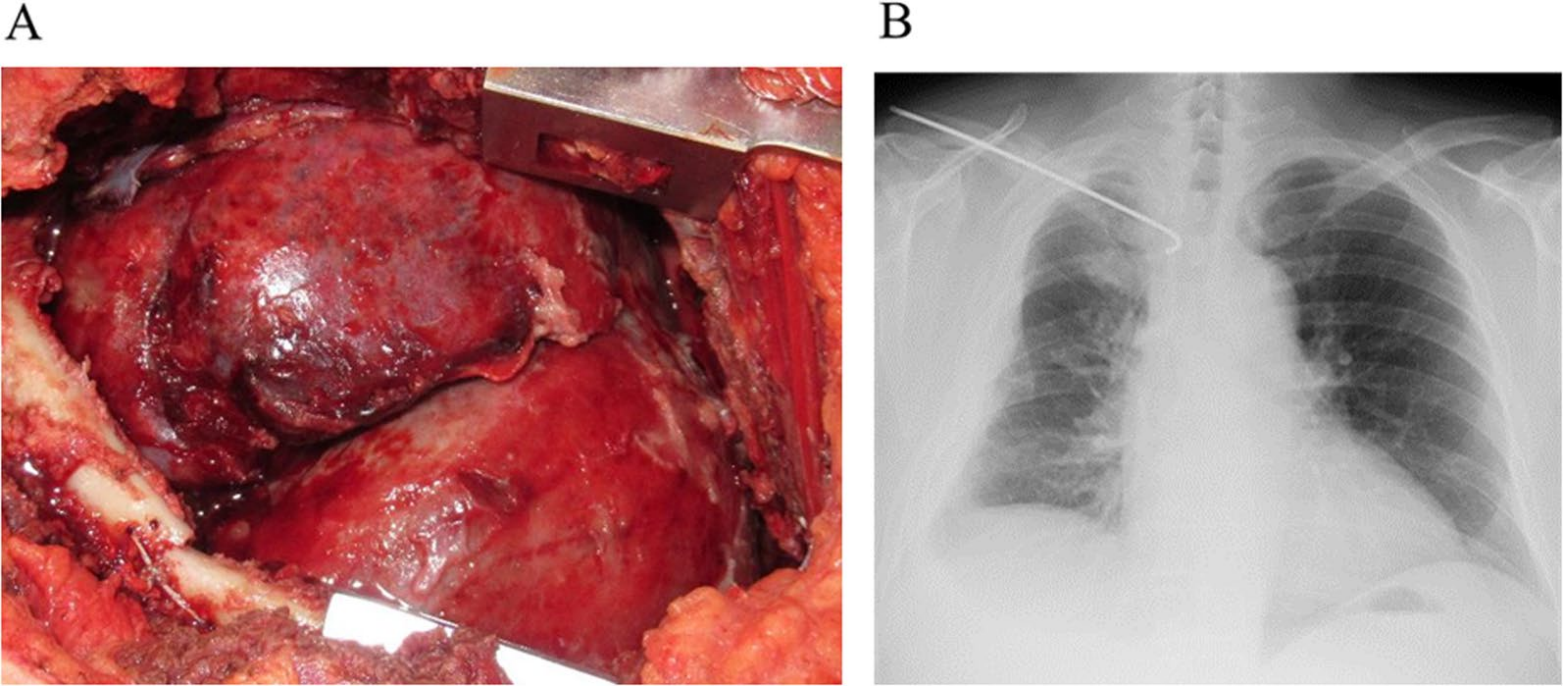
**A** The cavity of empyema with partial decortication after scraping and drainage. **B** Chest X-ray after surgery on postoperative day 16

Chest tube replacement and empyema irrigation with saline were performed while administering intravenous antibiotics. However, the condition of the unexpandable lung did not improve. On day 15 of hospitalization, radical surgery was performed via posterolateral thoracostomy for thoracic empyema and chest wall destruction caused by rib and clavicle fractures. The purulent fibrin clot firmly adhered to the visceral pleura, consistent with lung injury due to lung collapse. Therefore, the empyema cavity was scraped and drained with partial decortication. After adequate decortication, the collapsed lung showed full expansion and there was no lung fistula. The fractured ribs and clavicle were repaired and fixation without plates was performed (Fig. 3). Pleural fluid culture obtained during surgery was negative; thus, the causative bacteria could not be identified.

The postsurgical phase was uneventful and the patient was discharged on day 31. A 12-month follow-up did not reveal any significant findings.

## Discussion

Thoracic empyema that develops from thoracic injury, such as traumatic pneumothorax, can lead to life-threatening conditions, posing an important clinical issue [2]. However, published case reports in the PubMed database do not mention traumatic pneumothorax complicated by thoracic empyema. Therefore, this case highlights an important clinical issue. In our case, traumatic pneumothorax with rib fractures was treated with tube thoracostomy without antibiotic prophylaxis, but the case was complicated by thoracic empyema. The empyema was intractable, and the patient underwent radical surgery with decortication of the lung and chest wall repair with rib and clavicle fracture fixation.

In the present case, the possible main causes of the thoracic empyema included ineffective chest tube drainage for persistent pneumothorax. Other possible causes included progressive chest wall instability, and restricted ventilation failure due to obesity [8]. Although there was no instability at the time of presentation, rib and clavicular fractures morphology, and obesity could have caused the delayed chest instability. It was speculated that placement of the chest tube may have exacerbated the original rib fracture, leading to chest wall destruction. Some studies have reported complications after tube thoracotomy [9–11]. One study identified ineffective chest tube drainage, pulmonary contusion, and length of chest tube management as predictors of thoracic empyema after tube thoracotomy [11]. Based on the present findings, when persistent pneumothorax, multiple rib fractures including clavicle fractures, obesity, and long chest tube management are expected, management should be performed assuming empyema, including antibiotic prophylaxis administration.

The empyema was intractable, and the patient underwent radical surgery, while decortication of the lung was performed via posterolateral thoracostomy. The inflammatory state was more severe than expected for acute empyema, possibly due to pulmonary contusion and/or hemopneumothorax [8]. Thus, pulmonary contusions and hemopneumothorax complicated by infection may be intractable at an early stage. Plate fixation has become the mainstay of fixation for rib fractures, but the outcome of plate fixation in infected case remains controversial [12]. Therefore, chest wall repair with rib fixation was performed using suturing alone without plate fixation in our patient. Chest wall repair with suturing alone was sufficient for postoperative stabilization of the chest wall during short-term observation. In general, early surgery is recommended for cases of chest injury with chest wall instability and persistent pneumothorax [7, 13, 14]. In the present case, chest wall instability was not evident at the time of presentation, air leak was not persistent, and the patient was obese; therefore, the patient was treated conservatively, considering the risk of surgery. If there had been no chest wall instability or persistent pneumothorax, video-assisted thoracic surgery would have been considered. However, the chest wall instability worsened and was complicated by thoracic empyema; therefore, radical surgery was performed. In cases with risk factors, such as obesity, and signs of worsening, it may be better to consider antibiotic administration and surgery at an early stage due to the possibility of occult infection [3, 6, 13, 14].

The present case highlights the fact that traumatic pneumothorax can be complicated by empyema, which is a potentially intractable and life-threatening situation. As published papers indicate, antibiotic administration in tube thoracotomy can be recommended in select cases, such as persistent pneumothorax, pulmonary contusion, multiple rib fractures including clavicle fractures, obesity, and long chest tube management [3, 6], and early surgical intervention in thoracic injury can be effective if surgery is indicated [7, 13, 14]. When managing thoracic injury with pneumothorax and rib fractures, the identification of risk factors may help improve the patient’s prognosis. To ensure that the best strategy and tactics are adopted for such cases, further reporting of cases with similar physiological and anatomical findings is warranted.

## Conclusions

A traumatic pneumothorax can be complicated by empyema. Thoracic injuries complicated by empyema can be life-threatening and intractable. Antibiotic administration for traumatic pneumothorax with tube thoracotomy should therefore be considered in select cases. Surgery for thoracic injury with rib fracture and pneumothorax should be considered at an early stage, assuming an occult thoracic infection and chest wall instability.

## Data Availability

Not applicable.
